# A Dual-Polymer Fiber Fizeau Interferometer for Simultaneous Measurement of Relative Humidity and Temperature

**DOI:** 10.3390/s17112659

**Published:** 2017-11-17

**Authors:** Chao-Tsung Ma, Yu-Wei Chang, Yuan-Jie Yang, Cheng-Ling Lee

**Affiliations:** 1 Department of Electrical Engineering, National United University, Miaoli 36003, Taiwan; ctma@nuu.edu.tw; 2 Department of Electro-Optical Engineering, National United University, Miaoli 36003, Taiwan; as85635544@gmail.com (Y.-W.C.); nn4088nn40888@gmail.com (Y.-J.Y.)

**Keywords:** Fiber Fizeau interferometer (FFPI), polymer, fiber sensor, fast Fourier transform (FFT), simultaneously sensing humidity and temperature

## Abstract

This paper presents a novel design method in which a dual-polymer fiber Fizeau interferometer (DPFFI) is proposed for simultaneously measuring relative humidity (RH) and temperature (T). Since the polymer is intrinsically highly sensitive to both RH and T, the polymer fiber Fizeau interferometer (PFFI) exhibits cross-sensitivity of RH and T. In general, it is difficult to demodulate the optical responses from both variations of RH and T using a single PFFI. If two PFFIs with different structures are combined, they will individually exhibit distinct sensitivity responses with respect to RH and T, respectively. The technical problem of analyzing multiple interferences of the optical spectra of the DPFFI and the individual sensitivity of RH and T to each PFFI is obtained using the fast Fourier transform (FFT). A mathematical method is applied to solve the simultaneous equations of the DPFFI, so that the two variables RH and T can be determined at the same time. Experimental results, indicating good sensitivity and accuracy, with small measurement errors (average errors of ~1.46 °C and ~1.48%, respectively), are shown, determining the feasibility, and verifying the effectiveness, of the proposed DPFFI sensor.

## 1. Introduction

Relative humidity (RH) and temperature (T) are two significant physical parameters affecting a variety of industrial processes, such as semiconductor technology, food processing, biomedical engineering, weather forecasting, and environmental monitoring and control. In the above mentioned processes, the simultaneous measurement of RH and T provides a number of engineering advantages and is often a necessity for a variety of system applications. In recent years, several fiber optical sensors for simultaneous RH and T sensing have been proposed and investigated, but most of them are incorporated with well-known fiber devices such as fiber Bragg gratings (FBGs) [1,2,3,4,5,6,7,8], long-period fiber gratings (LPFGs) [8,9,10], and Fabry-Perot interferometers (FPIs) [1,11,12,13]. In [1], Arregui et al. proposed a sensor head composed of an FBG and a low-finesse FPI for simultaneous RH and T sensing. An RH measuring range from 11% to 97% RH and for T ranging from 10°C to 85 °C are shown. In their design case, the sensor head was connected to a single mode optical fiber network, which yielded a new possibility in multiplexing such sensors. An interesting fiber optic hybrid device composed of an FBG and a reflection-type photonic crystal fiber interferometer (PCFI) infiltrated with RH-sensitive agarose for simultaneous T and RH measurement was proposed and demonstrated in [2]. A measured T sensitivity of 9.8 pm/°C and an optical power variation of 7 dB upon a 75%RH change were shown. Another fiber device based on an FBG integrated with a photonic crystal fiber (PCF)-based in-fiber Mach-Zehnder interferometer (MZI) was developed in [3], where a short PCF was fusion-spliced between two single-mode fibers and coated with a layer of a polyvinyl alcohol (PVA) material. With the RH measurement range up to 30–95% RH, the simultaneous measurement has been achieved with resolutions of 1 °C and 0.13% RH for T and RH, respectively. A knob-integrated FBG was proposed in [4]. By exciting the cladding modes as well as recoupling the reflected cladding modes back into the leading single mode fiber, the proposed sensor reached an RH sensitivity of up to 1.2 dB/% RH within an RH range of 30–95% and a T sensitivity of 8.2 pm/°C in the T range of 25–60 °C. Massaroni C. et al. proposed a configuration based on an array of FBGs for achieving the simultaneous measurement of both T and RH of gas in a chamber [5]. Another sensor consisting of a Fabry–Perot cavity formed by two identical FBGs was proposed in [6], where the polyimide material was coated on the FBG and also on the cavity with a different thickness. RH and T sensitivities of 1.92 pm/% RH and 8.87 pm/°C, respectively, with coating thicknesses of 10 μm on the FBG and 15 μm on the cavity, were shown. A hygroscopic polymer microcavity fiber Fizeau interferometer (PMFFI) incorporating a fiber Bragg grating (FBG) was proposed in [7] for the simultaneous measurement of RH and T. High sensitivity and accuracy were achieved. A long-period fiber grating (LPG)-based sensing head with an in-line FBG was proposed in [8] for simultaneous measurement of RH and T. Measured resolutions of 1.6% RH and 2.5 °C were achieved within an RH range from 20 to 50%, while 2.4% RH and 0.4 °C resolutions were achieved within an RH range from 50 to 80%. A fiber loop mirror sensor with a long period grating (LPG) inscribed in the polarization maintaining fiber (PMF) was proposed in [9]. RH and T sensitivities of 0.1723 nm/% RH and 0.2174 nm/°C with measurement errors of 2.6% RH and 0.2 °C, respectively, were demonstrated. The authors of [10] compared the differences between the RH and T sensing capabilities of fully and half-coated LPGs and reported a simple and cost-efficient method of measuring both T and RH, a possible alternative to cascaded LPGs sensing systems. The main contribution of the proposed dual-wavelength-based sensing method is the simultaneous measurement of RH and T using only one LPG. Results showed sensitivities of 63.23 pm/% RH and 410.66 pm/°C for the attenuation band corresponding to the coated contribution, and 55.22 pm/% RH and 405.09 pm/°C for the attenuation band corresponding to the uncoated grating. In 2014, a dielectric multilayer-based fiber optic sensor with simultaneous RH and T measurement capability was proposed in [11]. Average RH and T sensitivities of 0.43 nm/% RH and 0.63 nm/°C, respectively, when environmental RH changes from 1.8% RH to 74.7% RH and T changes from 21.4 to 38.8 °C, with high repeatability were shown. In [12], an all-silica miniature fiber-optic sensor based on two cascaded FPIs, formed at the tip of an optical fiber to measure T and RH, was proposed. The authors of [13] proposed a sensing device using an optical fiber Fabry-Perot interferometer. That sensor was constructed by splicing a short length of PCF to a single-mode fiber and coating an ultrathin PVA film onto the PCF’s cleaved surface. However, of all the above-reviewed RH/T sensors, both the sensing sensitivity and resolution may not be high enough for certain high-tech applications. Furthermore, most of these fiber-grating-based sensors usually require complicated laser-written fabrication using expensive equipment.

In this work, simultaneous measurement of RH and T using a very simple and easily fabricated, dual-polymer fiber Fizeau interferometer (DPFFI) is presented. The polymer fiber Fizeau interferometer (PFFI) is based on a highly hygroscopic polymer directly coated on the end faces of two fibers. The presented hygroscopic polymer is highly sensitive to RH and T compared with previously reported fiber gratings. We strategically combined two PFFIs with different polymer lengths for the simultaneous measurement of RH and T. The key technical difficulty consisted in analyzing multiple interferences of optical spectra of the DPFFI and the individual RH and T sensitivities to each PFFI. By using the fast Fourier transform (FFT) and by solving a strategically simultaneous matrix inverse equation, the RH and T variables could be determined. Higher sensing sensitivities for RH and T were achieved, and no expensive equipment for laser-written fabrication of fiber-grating-based sensors was required. The proposed DPFFI device with design parameters of L_1_ = 43.92 μm and L_2_ = 12.5 μm was practically fabricated and tested under many different T/RH conditions. Experimental results show that the proposed DPFFI sensor is capable of effectively and simultaneously measuring the surrounding RH and T with good accuracy.

## 2. Configuration and Sensing Principle

**Figure 1:**
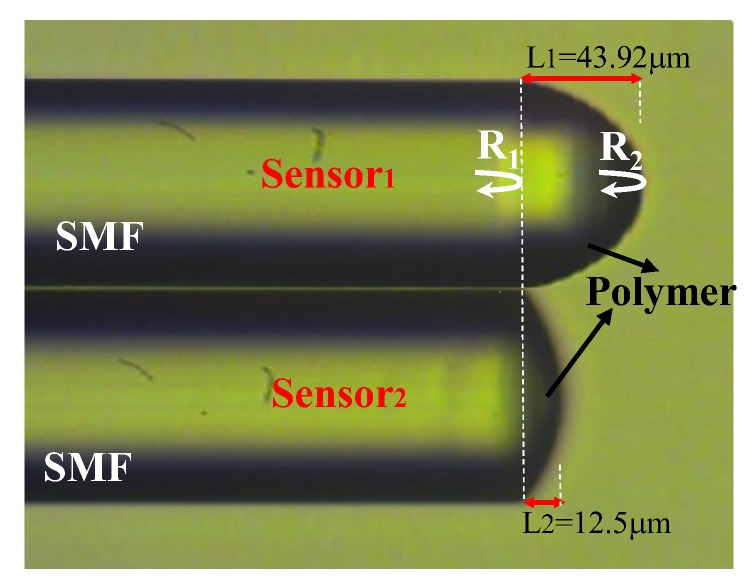
Configuration of the proposed dual-polymer fiber Fizeau interferometer (DPFFI) sensor.

**Figure 2:**
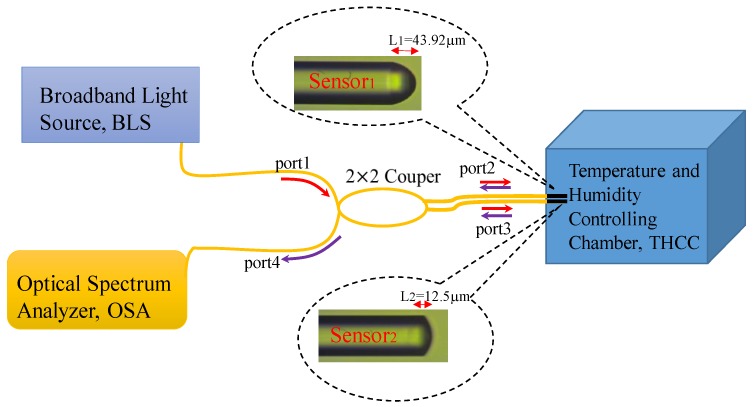
Experimental setup for simultaneously measuring RH and T.

The hygroscopic polymer Norland optical adhesive 61 (NOA61) is a photo polymerizable monomer that can be made into a solid polymer by ultraviolet (UV) light curing. We have presented a polymer microcavity fiber Fizeau interferometer incorporating an FBG for simultaneously sensing RH and T [7]. Here in this design, the FBG is not required; however, two hygroscopic polymer fiber Fizeau interferometers (PFFIs) with different cavities are strategically combined to form the proposed DPFFI sensor for achieving the simultaneous measurement of T and RH, as shown in Figure 1. Figure 1 shows the layer of the proposed polymer acting as a cavity with two reflective interfaces R_1_ and R_2_, which reflects the optical signals back into the single mode fiber again so that reflection interference is achieved by the fiber Fizeau interferometer. By using monitored translation stages, the thick film of the polymer can be attached to the fiber end face and the UV-curing can be applied to form the first PFFI (sensor_2_ in Figure 1). The coating and UV-curing steps can be repeated several times to obtain the second PPFI with a longer cavity length (sensor_1_ in Figure 1). The optical characteristics of the coating polymer on SMF varies in response to the RH or T of the surrounding changes, affecting the optical length of the Fizeau cavity as well as the optical phase difference between the two reflected beams. According to the results in [7], this hygroscopic polymer was intrinsically highly sensitive to RH as well as T, and the RH/T sensitivities therein depended on the lengths of the polymer cavity. Thus, by combining two PPFIs with different responses of RH and T, respectively, the proposed sensor was able to individually extract the variations of RH and T from the surrounding area. Figure 2 displays the experimental setup for performing the simultaneous measurement. The light signal from a broad band light source propagates with a 2 × 2 coupler, reflects off the endfaces of sensor_1_ and sensor_2_, and returns to the coupler again. Finally, the combined spectral response readouts can be directly obtained by an optical spectrum analyzer (OSA).

**Figure 3:**
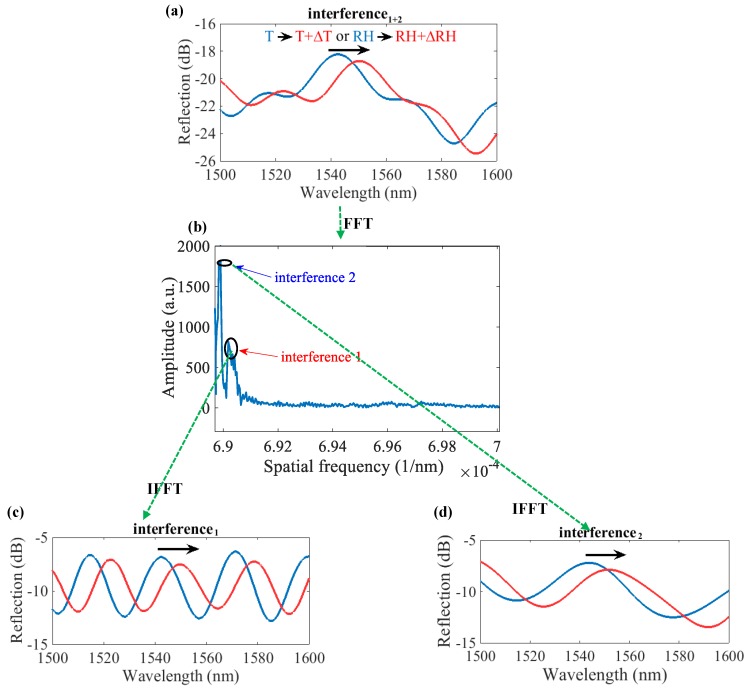
(**a**) Optical response of superimposed interference measured by the OSA; (**b**) superimposed spectra processed by FFT; separated optical spectra of (**c**) interference_1_ and (**d**) interference_2_, respectively.

Since we used two polymer sensors for the measurement of multiple parameters, the optical responses of the DPFFI are superimposed, will be displayed on the OSA. The analysis of the optical responses from the combined interferences can be accomplished using the fast Fourier transform (FFT) method. The FFT method was used to separate multiple interferences in spatial frequency into two individual spatial frequencies for sensor_1_ and sensor_2_, respectively. Figure 3 shows the processes of the optical response of the superimposed interference, separating into the spectra of interference_1_ and interference_2_ by the FFT method for sensor_1_ and sensor_2_, respectively. Figure 3a shows the multiple interferences of optical responses measured originally by the OSA. The spectral shifts are due to variations in the T and RH of the surrounding area, and the shifts that are caused by RH and/or T cannot be identified. Therefore, the signals of the superimposed interference of Figure 3a are processed by the FFT to obtain spatial frequency spectra, as shown in Figure 3b. We can then individually reconstruct interference_1_ and interference_2_ for sensor_1_ and sensor_2_ by the inverse fast Fourier transform (IFFT), as plotted in Figure 3c,d, respectively. Then, in an identical variation of T or RH, wavelength shifts of the optical interference_1_ and interference_2_ for sensor_1_ and sensor_2_ can be independently determined for achieving the simultaneous measurement of RH and T. In Figure 3c, the optical response of interference_1_ can be inferred from the long-cavity of sensor_1_ since high spatial frequency comes from the long-cavity in sensor_1_. It can also be predicted that the sensitivities of RH and T in the long-cavity sensor_1_ are smaller than those obtained by the short-cavity sensor_2_.

If there are changes in temperature (∆*T*) and relative humidity (∆*RH*), wavelength shifts both from sensor_1_ (∆*λ*_1_) and sensor_2_ (∆*λ*_2_) can be respectively estimated using the simultaneous Equation (1a,b). In mathematics, a system of linear equations is a collection of two or more linear equations involving the same set of variables. Thus, a solution with several variables to a linear system can be obtained in which all the equations are simultaneously satisfied. Thus, by solving the simultaneous Equation (1a,b) of the sensing system, one can determine two variables (∆*T* and ∆*RH*) at the same time.

(1a) $$\frac{∆\lambda_{1}}{\lambda_{1}}=\frac{1}{d_{1}}\frac{\partial d_{1}}{\partial T}∆T+B∆RH=A∆T+B∆RH.$$

(1b) $$\frac{∆\lambda_{2}}{\lambda_{2}}=\frac{1}{d_{2}}\frac{\partial d_{2}}{\partial T}∆T+D∆RH=C∆T+D∆RH.$$

In Equation (1a,b), *A* and *C* as well as *B* and *D* are the corresponding T and RH sensitivity coefficients for sensor_1_ and sensor_2_, respectively. Here, *d*_1_ and *d*_2_ denote the lengths of the polymer cavity for sensor_1_ and sensor_2_, respectively. The coefficients *A*, *B*, *C*, and *D* can be determined by measuring the individual spectral sensitivities of the two PFFIs in the DPFFI system with the variations in *T* and RH. Then, using the matrix inversion method, variations in temperature (∆*T*) and relative humidity (∆*RH*) can be simultaneously obtained by measuring the wavelength shifts of PFFI_1_ (∆*λ*_1_) and PFFI_2_ (∆*λ*_2_), respectively. The relation of the above parameters according to the Equation (1) is described below:

(2) $$\left(\begin{array}{c} \begin{array}{l} ∆T \end{array}\\ ∆RH \end{array}\right)={\left(\begin{array}{cc} A & B\\ C & D \end{array}\right)}^{-1}\left(\begin{array}{c} \begin{array}{l} \frac{∆\lambda_{1}}{\lambda_{1}} \end{array}\\ \frac{∆\lambda_{2}}{\lambda_{2}} \end{array}\right).$$

Equation (2) can be simplified as follows:

(3) $$\left(\begin{array}{c} \begin{array}{l} ∆T \end{array}\\ ∆RH \end{array}\right)={\left(\begin{array}{cc} A\prime & B\prime \\ C\prime & D\prime \end{array}\right)}^{-1}\left(\begin{array}{c} \begin{array}{l} ∆\lambda_{1} \end{array}\\ ∆\lambda_{2} \end{array}\right)$$

where *A*′, *C*′, *B*′, and *D*′ represent the normalized coefficients for the T and RH sensitivities of sensor_1_ and sensor_2_, respectively. As a result, the sensitivity coefficients *A*′, *C*′, *B*′, and *D*′ in the sensing configuration need to be evaluated primarily to achieve the simultaneous measurement of RH and T.

## 3. Experimental Results and Discussion

**Figure 4:**
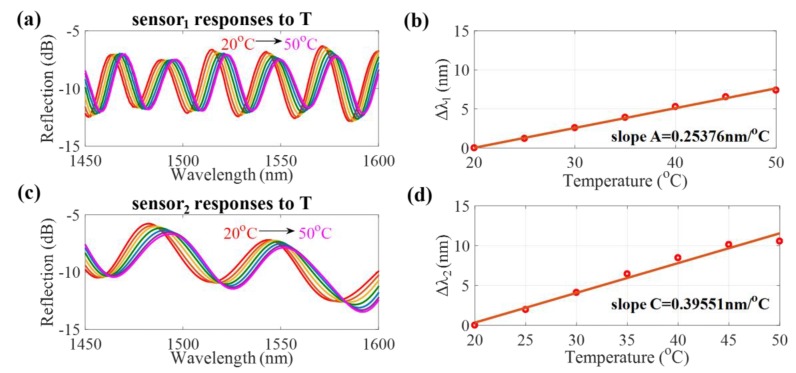
(**a**,**c**) Interference spectra of sensor_1_ and sensor_2_ for different T values, respectively; (**b**,**d**) Corresponding T sensitivities for sensor_1_ and sensor_2_, respectively.

**Figure 5:**
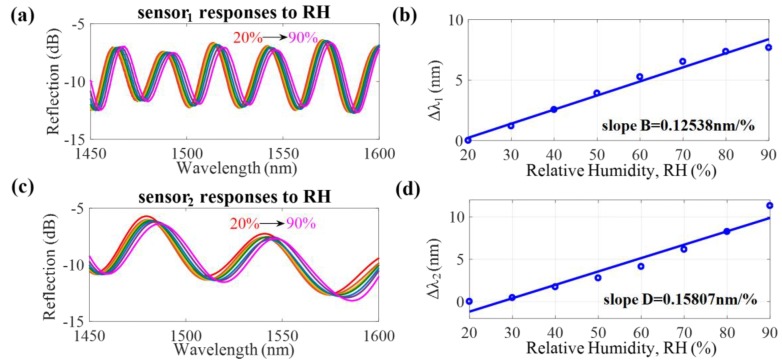
(**a**,**c**) Interference spectra of sensor_1_ and sensor_2_ for different RH values, respectively; (**b**,**d**) Corresponding RH sensitivities for sensor_1_ and sensor_2_, respectively.

In the experiment, the proposed DPFFI device was placed inside a temperature and humidity controlling chamber (THCC), as displayed in Figure 2, which was a closed space in which the T was increased from 20 to 50 °C and RH was fixed. The measured results are shown in Figure 4. The spectral responses to T are displayed in Figure 4a,c for sensor_1_ and sensor_2_, respectively. Based on the individual responses of interference_1_ and interference_2_ of sensor_1_ and sensor_2_, the T sensitivities of sensor_1_ and sensor_2_ to T are +0.25376 nm/°C and +0.39551 nm/°C, respectively, plotted in Figure 4b,d. Again, the device was placed in a condition where RH was increased from 20 to 90% and T was fixed at 25 °C. Figure 5 shows the RH sensitivities of sensor_1_ and sensor_2_: +0.12538 nm/%RH and +0.15807 nm/%RH, respectively. These results demonstrate that the proposed sensor has a wavelength redshift response when either T or RH is increased. It can also be inferred that shorter cavity lengths have larger free spectral ranges (FSRs) with greater T and RH sensitivities. However, it could be that, because of the possible overlap of interference dips, the measurement range is limited by the length of the FSR. The amount of wavelength shifts approaching the FSR will lead to the overlap of interference signals, so the wavelength shifts cannot be well identified. To avoid an overlap of interference dips, appropriate ranges of T (20~50 °C) and RH (20~90%) were considered in our study to obtain the T and RH sensitivity slopes and to demonstrate the proposed sensing method. 

Based on the above experimental results, the normalized sensitivity parameters *A*′, *B*′, *C*′, and *D*′ are the T and RH sensitivity coefficients of sensor_1_ and sensor_2_, which are estimated to be 0.25376 nm/°C, 0.12538 nm/%RH, 0.39551 nm/°C, and 0.15807 nm/%RH, respectively. The matrix inversion method is then utilized to perform the simultaneous measurement of RH and T. The analytical simultaneous equation, Equation (3), can be expressed as Equation (4). The inverse matrix can then be used to simplify Equation (4), yielding Equation (5).

(4) $$\left(\begin{array}{c} ∆T\\ ∆RH \end{array}\right)={\left(\begin{array}{cc} A^{\prime } & B^{\prime }\\ C^{\prime } & D^{\prime } \end{array}\right)}^{-1}\left(\begin{array}{c} ∆\lambda_{1}\\ ∆\lambda_{2} \end{array}\right)={\left(\begin{array}{cc} 0.25376 & 0.12538\\ 0.39551 & 0.15807 \end{array}\right)}^{-1}\left(\begin{array}{c} ∆\lambda_{1}\\ ∆\lambda_{2} \end{array}\right).$$

(5) $$\left(\begin{array}{c} ∆T\\ ∆RH \end{array}\right)=\frac{1}{-9.4914\times 10^{-3}}\left(\begin{array}{cc} 0.25376 & -0.39551\\ -0.12538 & 0.15807 \end{array}\right)\left(\begin{array}{c} ∆\lambda_{1}\\ ∆\lambda_{2} \end{array}\right).$$

**Figure 6:**
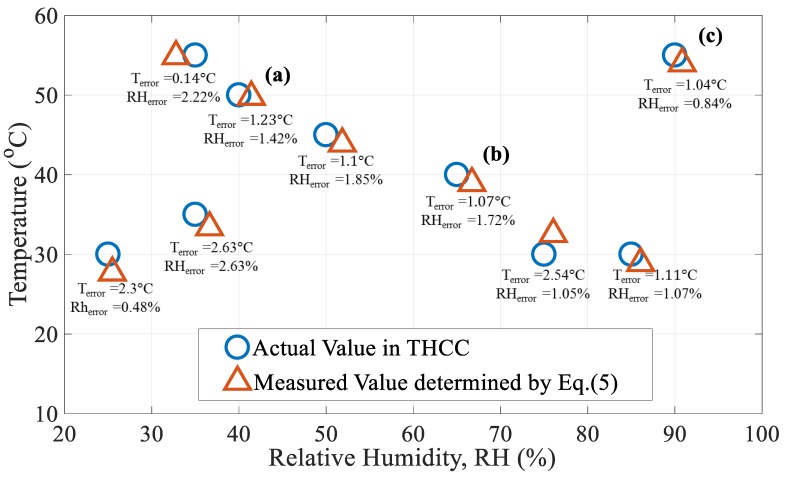
Sample data for measurement comparison.

**Figure 7:**
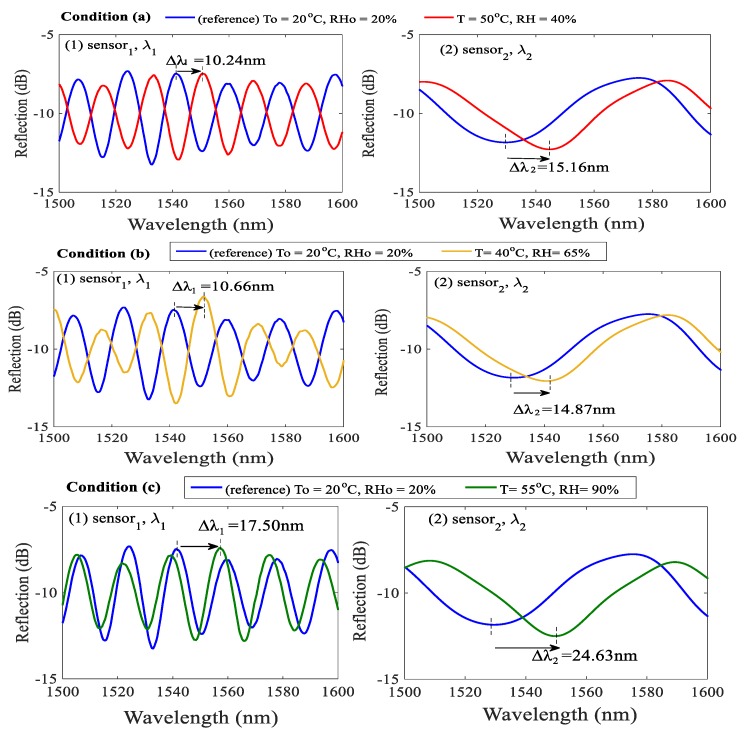
(1) and (2) are interference spectra shifts of sensor_1_ and sensor_2_, respectively, when RH and T change simultaneously in Conditions (**a**–**c**).

To investigate the effectiveness of Equation (5) for the developed sensing configuration, T and RH were simultaneously varied from their reference values of ambient T_0_ = 20 °C and RH_0_ = 20% to conditions shown in Figure 6 and Figure 7. The optical spectra of the initial condition of T_0_ = 20 °C and RH_0_ = 20% for sensor_1_ and sensor_2_ were recorded at the beginning of the experimental tests (The blue line in Figure 7). The Figure 6 presents the simultaneous measurement of random T and RH values under various levels. The blue circles represent the states of the T and RH levels in the temperature and humidity controlling chamber (THCC). The red triangles are the measured data evaluated by the proposed method. One can see that, in Figure 6, the simultaneous sensing of T and RH was accomplished. The average errors of the T and RH measurements in Figure 6 are about ~1.46 °C and ~1.48 %, respectively. We think that the errors are attributable to the measured deviations of the THCC machine and errors of numerical calculation. The results of three randomly selected cases—(a) T = 50 °C, RH = 40%, (b) T = 40 °C, RH = 65%, and (c) T = 55 °C, RH = 90% (marked in Figure 6)—whose measured spectral data and optical wavelength shifts are shown in Figure 7 and evaluated in Table 1, respectively. 

In Condition (a) of Figure 7 as well as in Table 1, the measured wavelength shifts of sensor_1_ and sensor_2_ are ∆λ_1_= 10.24 nm and ∆λ_2_=15.16 nm, respectively. Based on Equation (5), the changes in T and RH are readily obtained as ∆T = +29.77 °C and ∆RH = +21.42%, respectively. Thus, the measurements of T and RH in the Condition (a) are T_m_ = 20 + 29.77 = 49.77 °C and RH_m_ = 20 + 21.42 = 41.42%, respectively. The simultaneously measured values of T_m_ and RH_m_ are very similar to the actual values displayed in the THCC. The errors in T and RH are 0.23 °C and 1.42%, respectively. The other two measurements of Conditions (b) and (c) with measured values of ∆λ_1_ = 10.66 nm and ∆λ_2_ = 14.87 nm, and ∆λ_1_ = 17.50 nm and ∆λ_2_ = 24.63 nm, used to determine T_m_ and RH_m_, are also shown in Table 1 and Figure 7. The above evaluation demonstrates that effectiveness of the proposed DPFFI sensor.

## 4. Conclusions

We have developed a dual polymer fiber Fizeau interferometer (DPFFI) and used it to measure relative humidity (RH) and temperature (T) simultaneously using the fast Fourier transform (FFT) and a strategically mathematical matrix inversion relation. Since the proposed PFFI is highly sensitive to T and RH, sensing configuration of the developed DPFFI strategically combined with two different PFFIs can individually identify the variations in T and RH to achieve the objective of simultaneous measurement. The sensing principle is based on the fact that both PFFIs exhibit diverse RH and T sensitivities, respectively. Comprehensive experimental tests have been accomplished. Consistent results show that, by using the proposed sensing scheme with a simple mathematical method, the proposed DPFFI can simultaneously and effectively measure T and RH with small errors in sensing ranges of T = 20~50 °C and RH = 20~90%.

## Figures and Tables

**Table 1:** Evaluating the simultaneous measurement of T and RH variations in different conditions of the proposed sensor.

	Condition	(a) T = 50 °C, RH = 40%	(b) T = 40 °C, RH = 65%	(c) T = 55 °C, RH = 90%
Parameter				
Δλ_1_ (nm)		+10.24	+10.66	+17.50
Δλ_2_ (nm)		+15.16	+14.87	+24.63
Determined by Equation (5)		ΔT = + 29.77 °C	ΔT = +18.93 °C	ΔT = +33.96 °C
		ΔRH = + 21.42%	ΔRH = +46.72%	ΔRH = +70.84%
Measured T_m_ and RH_m_		T_m_ = T_0_ + ΔT = 49.77 °C	T_m_ = T_0_ + ΔT = 38.93 °C	T_m_ = T_0_ + ΔT = 53.96 °C
		RH_m_ = RH_0_ + ΔRH = 41.42%	RH_m_ = RH_0_ + ΔRH = 66.72%	RH_m_ = RH_0_ + ΔRH = 90.84%
T_error_ = |T_m_ − T|		T_error_ = 0.23 °C	T_error_ = 1.07 °C	T_error_ = 1.04 °C
RH_err__or_ = |RH_m_ − RH|		RH_error_ = 1.42%	RH_error_ = 1.72%	RH_error_ = 0.84%
